# Editorial for the Special Issue “Neurobiology of Invertebrates Inaccessible from Mammalian Studies”

**DOI:** 10.3390/biology14030290

**Published:** 2025-03-13

**Authors:** Etsuro Ito, Kengo Namiki

**Affiliations:** Department of Biology, Waseda University, Tokyo 162-0056, Japan; k-namiki38295k2r@ruri.waseda.jp

This Special Issue features five excellent papers on invertebrate neurobiology. We strongly believe that neurobiological research using invertebrates is vitally important, with further developments being anticipated in the future. Studies using invertebrates are highly advantageous for a number of reasons: (a) Simpler nervous systems: Invertebrates have far fewer neurons than vertebrates, facilitating studies of individual neurons and their connections and simplifying the process of understanding how neural circuits work. Taking advantage of the open vasculature and controlling specific behaviors with just one or a few neurons has yielded excellent results that could not have been obtained in mammalian studies. (b) Large, identifiable neurons: Some invertebrates, such as the pond snail *Lymnaea*, have neurons that are exceptionally large and easy to identify, making them ideal for studying the properties of individual neurons and their interneuronal communication. (c) Ease of breeding: Invertebrates are easy to raise, are generally low-maintenance, and have readily available food sources. (d) Ethical considerations: Invertebrates are generally considered to have simpler cognitive abilities than vertebrates, raising fewer ethical concerns about their use in research. (e) Evolutionary insights: Studying the nervous systems of invertebrates can provide insights into evolutional changes in nervous systems. (f) Genetic advantages: Some invertebrates have few homologs or orthologs. In addition, some invertebrates, such as fruit flies (*Drosophila*) and worms (*C. elegans*), have been extensively studied genetically, making it possible to use genetic tools to manipulate and study their nervous system in ways that are not possible in vertebrates. Overall, invertebrates serve as valuable models for neurobiological studies. Their advantages make them ideal for addressing a wide range of research questions.

First, we introduce three neurobiological studies using the pond snail *Lymnaea stagnalis*. Insulin and insulin-like peptides contribute to improving learning and memory in both vertebrates and invertebrates. Adiponectin has blood-glucose-lowering and insulin-sensitivity-increasing effects in mammals. These two facts led Fujimoto and colleagues to hypothesize that adiponectin and its receptors play an important role in learning and memory [1]. They tested this hypothesis using *L. stagnalis*. Genes coding the putative molecules of adiponectin and its receptor in *L. stagnalis* were identified, and their localization in the central nervous system and changes in their expression levels were examined under various nutritional conditions. Next, an operant conditioning protocol of escape behavior was applied to the snails, and changes in the expression levels of adiponectin and its receptor were investigated. Adiponectin was upregulated by food deprivation, whereas the expression of its receptor was upregulated after operant conditioning was established. These findings suggest the involvement of the adiponectin-signaling cascade in learning and memory in *L. stagnalis* through changes in the concentrations of glucose and the activation of insulin.

Chistopolsky and colleagues examined whether intense locomotion enhances oviposition in *L. stagnalis* [2]. It remains largely unknown if reproductive behavior is affected by physical activity (exercise). *L. stagnalis* can be used to investigate events at the molecular and cellular levels. The authors found that intense crawling in shallow water (exercise) for 2 h resulted in an increased number of egg clutches and the total number of eggs laid in the following 24 h. This effect was stronger from January to May compared with September to December. The synthesis of RNA transcripts of the egg-laying hormone was higher in the central nervous system of snails that exercised. Additionally, the caudodorsal neurons, which produce the egg-laying hormone and play a key role in egg-laying behavior, responded more strongly to electrical stimulation in exercised snails. Their findings suggest that exercise enhances reproduction in *L. stagnalis* despite its obvious energetic costs. These data support the hypothesis that the behavioral effects of exercise emerged early in evolution and have adaptive significance: preparing an organism to enter a new environment.

Rivi et al. studied the Garcia effect [3]. The Garcia effect, also known as conditioned taste aversion, occurs when an animal develops a strong aversion to a particular food or taste after experiencing nausea following its consumption. This aversion occurs even if the food itself was not the cause of the sickness. Using *L. stagnalis* in a Garcia effect training procedure, they studied novel aspects of this complex and highly conserved conditioned behavior and its pharmacologic regulation. Injecting snails with lipopolysaccharide (LPS) after they experienced a novel taste caused snails to form a long-lasting Garcia effect memory to avoid that specific taste. The effect was prevented by pre-exposing snails to acetylsalicylic acid (ASA) before the LPS injection. Here, they researched the transcriptional effects of ASA and LPS in the snails’ central nervous system, both separately and in combination, as well as in naive snails. In a similar manner, the behavioral and molecular mechanisms causing the LPS-induced Garcia effect and its mitigation by ASA were studied. The LPS injections enhanced the expression levels of immune and stress response targets, and enhancement was prevented by pre-exposure to ASA. LPS alone did not affect the expression levels of genes associated with neuroplasticity. When combined with the Garcia effect training procedure, however, the gene expression levels were upregulated, consistent with long-term memory formation. These findings suggest conserved crosstalk between the immune and central nervous systems. *L. stagnalis* is widely used in various aspects of learning and memory research [4].

A study using the marine spionid *Pygospio elegans* is also included [5]. Shunkina and colleagues studied how *P. elegans* regenerates its nervous system at the anterior and posterior ends. They used immunostaining techniques with antibodies against serotonin and FMRFamide to label the nervous system of intact and regenerating worms. Their findings showed that regeneration of the *P. elegans* central nervous system has common features with that of other annelids, whereas regeneration of the peripheral nervous system depends on individual features. Comparing these findings with the results for other annelids provides valuable insights into both conservation and plasticity in the mechanisms of nervous system regeneration.

The final study by Kotsyuba et al. used the king crab *Paralithodes camtschaticus* [6]. This study provides the first description of the pattern of central nervous system development in red king crabs, particularly the changes that occur during the two metamorphoses of their free-swimming planktonic larvae. The results of the study led to the creation of a map of the neural architecture and distribution of cells producing serotonin and dopamine in the medial brain and ventral nerve cord of the larval nervous system, which provides insights into the potential functions of serotonin and dopamine, both in red king crab larvae at all developmental stages and in adults.

We expect that neurobiological research using invertebrates will continue to develop in the future, providing significant insights into the basics of biology, which will ultimately lead to a greater understanding of humans.
